# A simple strategy for retargeting lentiviral vectors to desired cell types via a disulfide-bond-forming protein-peptide pair

**DOI:** 10.1038/s41598-018-29253-5

**Published:** 2018-07-20

**Authors:** Nagarjun Kasaraneni, Ana M. Chamoun-Emanuelli, Gus A. Wright, Zhilei Chen

**Affiliations:** 1 Department of Microbial Pathogenesis and Immunology, Texas A&M University Health Science Center, College Station, Texas 77843 USA; 2 Department of Veterinary Pathobiology, Texas A&M University, College Station, TX 77843 USA

## Abstract

Despite recent improvements in the engineering of viral envelope proteins, it remains a significant challenge to create lentiviral vectors that allow targeted transduction to specific cell populations of interest. In this study, we developed a simple ‘plug and play’ strategy to retarget lentiviral vectors to any desired cell types through *in vitro* covalent modification of the virions with specific cell-targeting proteins (CTPs). This strategy exploits a disulfide bond-forming protein-peptide pair PDZ1 and its pentapeptide ligand (ThrGluPheCysAla, TEFCA). PDZ1 was incorporated into an engineered Sindbis virus envelope protein (Sind-PDZ1) and displayed on lentiviral particles while the TEFCA pentapeptide ligand was genetically linked to the CTP. Her2/neu-binding designed ankyrin repeat proteins (DARPin) were used as our model CTPs. DARPin-functionalized unconcentrated lentiviral vectors harboring Sind-PDZ1 envelope protein (Sind-PDZ1-pp) exhibited >800-fold higher infectious titer in HER2^+^ cells than the unfunctionalized virions (8.5 × 10^6^ vs. <10^4^ IU/mL). Moreover, by virtue of the covalent disulfide bond interaction between PDZ1 and TEFCA, the association of the CTP with the virions is nonreversible under non-reducing conditions (e.g. serum), making these functionalized virions potentially stable in an *in vivo* setting.

## Introduction

Gene therapy has the potential to treat any genetically caused disease including monogenetic disorders and cancers. A significant barrier to gene therapy is specific delivery of the genetic material in sufficient quantities to the target cells to achieve a therapeutic effect. Viruses are natural gene delivery vehicles and have been extensively exploited as gene therapy vectors^1^. In particular, lentiviral vectors engineered from human immunodeficiency virus (HIV) are capable of efficient gene delivery to both mitotic and nondividing cells^2^, and have emerged as a promising and apparently safe vehicle for clinical gene therapy. Lentiviral vectors integrate into the host cell genome and thus are duplicated along with the host DNA during mitosis, enabling long-term transgene expression. The recent FDA approval of Kymriah (CTL019)^3^, which creates CAR T-cells against CD19 receptor for treating a form of acute lymphoblastic leukemia (ALL), highlights the potential of lentiviral vectors in gene therapy. Kymriah and most other existing lentiviral vector-based gene therapies however, rely on *ex vivo* gene delivery as lentiviruses pseudotyped with vesicular stomatitis virus glycoprotein (VSV-Gpp), the most commonly used envelope protein, were found to be rapidly neutralized by serum complement^4,5^. In addition, vesicular stomatitis virus enters cells through the LDL family of receptors that are nearly omnipresent^6^, rendering VSV-Gpp promiscuous to a wide range of cells. The broad tropism of VSV-Gpp makes it an non-ideal *in vivo* gene therapy vector as it is often necessary to restrict the delivery of the therapeutic gene to only the desired type to minimize cytotoxicity^7^.

Several strategies have been developed to create cell-specific lentiviral vectors^8^. One common strategy is to incorporate envelope glycoproteins derived from different viruses (pseudotyping)^9^. However, natural viral envelope proteins are often poorly specific for clinically relevant cell-types. In addition, significant modification of the foreign glycoprotein’s cytoplasmic region is often needed to enable efficient pseudotyping^9–11^. Another strategy is to incorporate new cell targeting proteins (CTPs) into the outer surface of the virus envelope. Entry of enveloped viruses into cells involves two major steps: virus-cell attachment and fusion of viral and cellular membrane. Fortunately, for many viruses, these two steps function independently. For viruses with an abolished wild type attachment function (blinded envelope protein), incorporation of a new CTP can retarget the virus. A prominent strategy to incorporate new CTPs into viruses is by fusing the CTP directly to the viral envelope protein^12^. For example, Buchholz and co-workers reported the creation of lentiviral vectors specific for different cell types through fusion of different cell-targeting proteins to a binding-deficient fusion-competent Nipah virus (NiV) envelope protein or Measles virus (MV) envelope protein and pseudotyping lentivirus with this new chimeric protein^13,14^. Some CTPs however, cannot be genetically incorporated into viruses using recombinant approaches due to “surface incompatibility”, limiting the types of cells accessible for gene therapy^15^.

**Figure 1:**
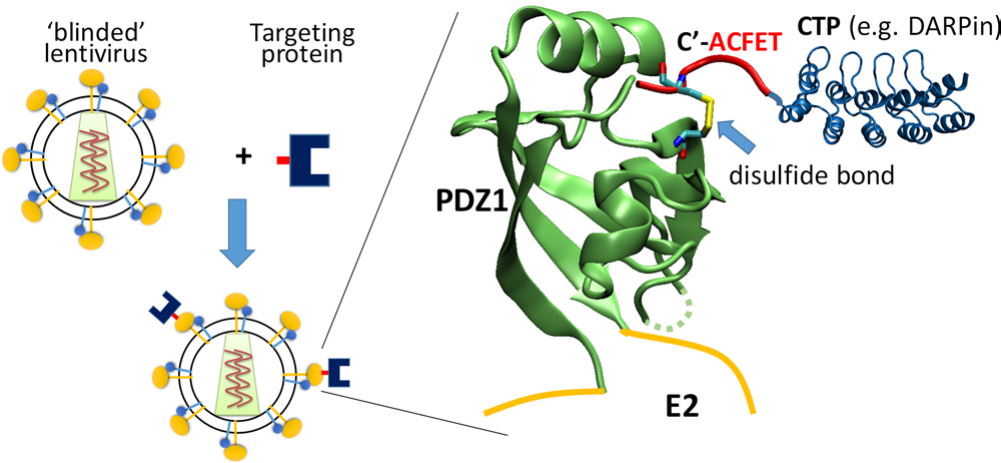
Scheme of our ‘plug-and-play’ lentiviral retargeting strategy. PDZ1 (PDB ID: 1ihj^19^) is inserted into a binding-deficient fusion-competent Sindbis virus E2 envelope protein and used to pseudotype lentivirus. The CTP (e.g. DARPin, PDB ID: 4j7w^35^) is fused to a short pentapeptide ligand (TEFCA). Mixing of these two components enables covalent functionalization of the lentivirus with the CTP through the formation of a disulfide bond between the PDZ1 and TEFCA ligand.

Previously, our lab developed a split-intein-mediated approach to retarget lentivirus^16^. That approach exploited a splicing-deficient variant of the naturally split intein from *Nostoc punctiforme*^17^. One half of the split intein – NpuN – was fused to a CTP, while the other half – NpuC*– was displayed on lentiviral vectors as fusion to a binding-deficient, fusion-competent Sindbis virus envelope protein to form Sind-C*^18^. The split intein functioned as a molecular Velcro linking the cell-binding protein to the pseudotyped lentivirus. Unfortunately, despite the low nanomolar affinity between the two halves of the split intein, the cell-targeting protein gradually dissociated from the virus during extended periods of dialysis due to the non-covalent interaction between the two halves of the split intein, reducing the specific transduction efficiency of the virus over time. In this study, we replaced the intein system with a covalent-bond-forming protein-peptide pair from the *Drosophila* visual system – the N-terminal PDZ domain of InaD protein (PDZ1) and its pentapeptide ligand (TEFCA) from NorpA^19^ – to conjugate a CTP to the lentiviral vector. The PDZ1 was inserted into a previously engineered binding-deficient, fusion-competent Sindbis virus E2 envelope protein between residue 71 and 74^18,20^ to form Sind-PDZ1 while the TEFCA tag was fused to a CTP. Specific interaction between PDZ1 and TEFCA enables lentiviral vectors displaying Sind-PDZ1 (Sind-PDZ1-pp) to be covalently functionalized (via a disulfide bond) with the desired CTP and retargeted to the desired cell type (Fig. 1).

Using a panel of HER2-binding designed ankyrin repeat proteins (DARPin.X)^21^ as our model CTPs, we created series of DARPin.X-TEFCA fusion proteins. These DARPin-functionalized Sind-PDZ1-pp efficiently transduced HER2^+^ SKOV3 cells with up to 8.5 × 10^6^ IU/mL (unconcentrated supernatant), >800-fold more efficiently than the unfunctionalized “naked” virions (<10^4^ IU/mL). The association of DARPin.X-TEFCA and Sind-PDZ1-pp appears to be non-reversible under non-reducing conditions owing to the covalent disulfide interaction between PDZ1 and TEFCA. Moreover, our functionalized virions retained full infectivity in the presence of human serum, supporting the use of these retargeted virions for *in vivo* gene therapy applications.

## Results

### Retargeting of lentivirus with DARPin-TEFCA

**Figure 2:**
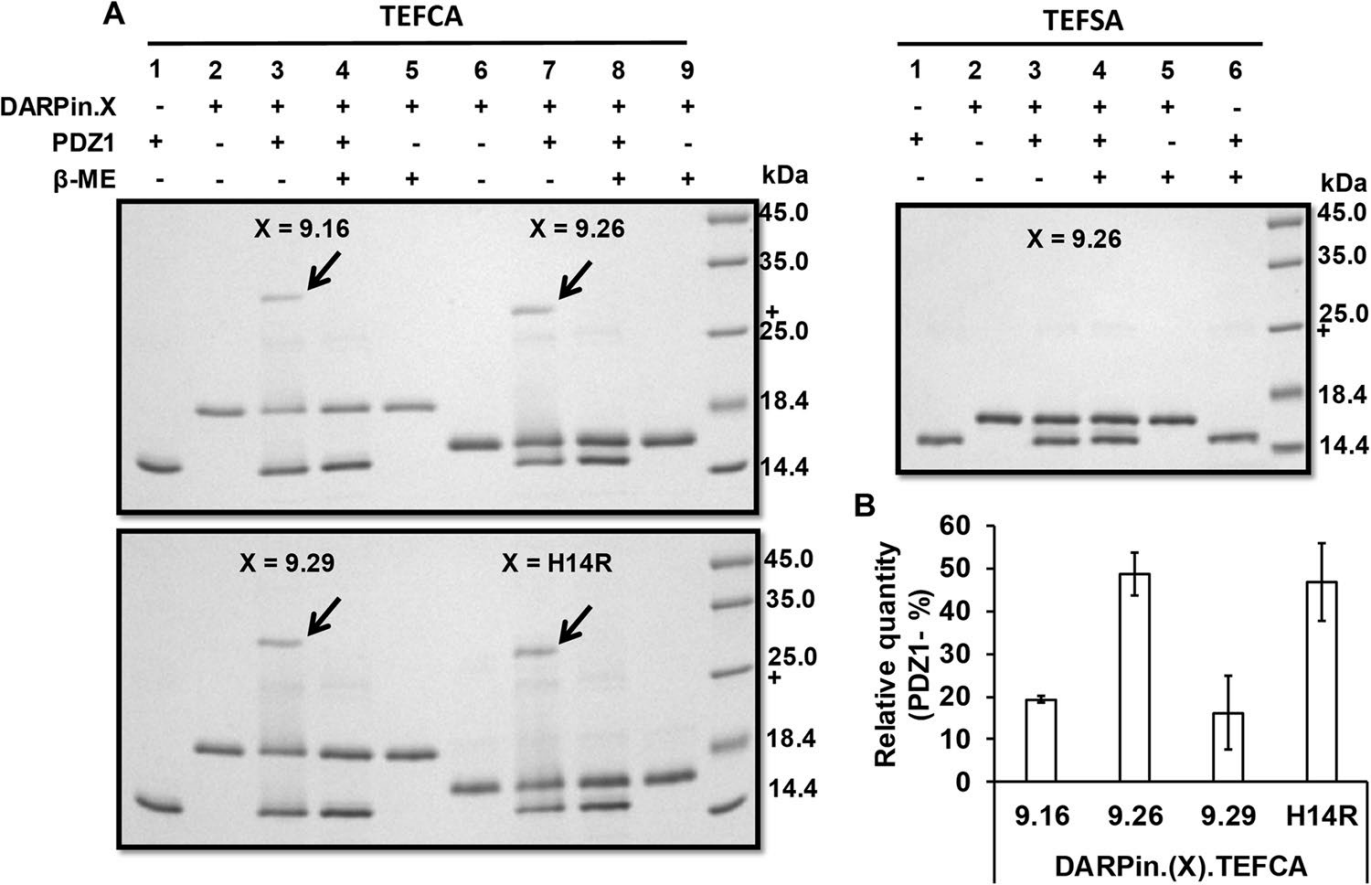
*In vitro* conjugation of DARPin.X-TEFCA and PDZ1. (**A**) PDZ1 (100 µM) was incubated with different DARPin.X-TEFCA constructs or the negative control DARPin.9.26-TEFSA (100 μM) at room temperature for 4 h. The mixture was then separated on 12% SDS-PAGE gels under reducing or non-reducing conditions. The conjugation products (indicated by black arrows) can only be seen under non-reducing condition (lane 3, 7). (**B**) The percentage of PDZ1 reacted with different DARPin.X.TEFCA was calculated by dividing the intensity of unreacted PDZ1 band in lane 3 or 7 with that in lane 1. Values and error bars represent the average and standard deviation, respectively, of at least two independent experiments.

We present a new strategy for retargeting lentivirus using a covalent-bond-forming protein-peptide pair: N-terminal PDZ domain of InaD protein (PDZ1) and its pentapeptide ligand (TEFCA) from NorpA^19^ (Fig. 1). Proteins containing a C-terminal TEFCA tag can be selectively pulled-down by PDZ1-functionalized resin with a K_ON_ >500 M^−1^s^−1^ under non-reducing conditions (the K_OFF_ is undefined due to the covalent linkage)^22^. We demonstrate our retargeting strategy using a panel of HER2/neu-binding designed ankyrin repeat proteins (DARPin.X)^23,24^ as our model CTPs. DARPins are a versatile class of binding proteins that have been engineered to bind diverse targets with up to picomolar affinity^25^. We first fused the TEFCA tag to these DARPins and confirmed the ability of the fusion proteins to form a disulfide bond with purified PDZ1 *in vitro* (Fig. 2A). Surprisingly, despite sharing the same overall structure, the efficiency of disulfide bond formation was quite different for the different DARPins (Fig. 2B). For DARPin.9.26 and H14R, ~50% of the input PDZ1 protein formed disulfide complexes with these DARPins after 4 h incubation at room temperature, while <20% of PDZ1 formed disulfide bonds with DARPin.9.16 and 9.29 under the same condition. It is unclear what caused the different disulfide bond formation efficiency, as the TEFCA tag was fused to the C-terminus of each DARPin via the same GGGG linker. As a negative control, we mutated the Cys in TEFCA to Ser to form TEFSA penta-peptide, and fused it to DARPin.9.26. No conjugation product was formed between DARPin.9.26-TEFSA and PDZ1 (Fig. 2A), confirming that the Cys in TEFCA is necessary for disulfide bond formation.

**Figure 3:**
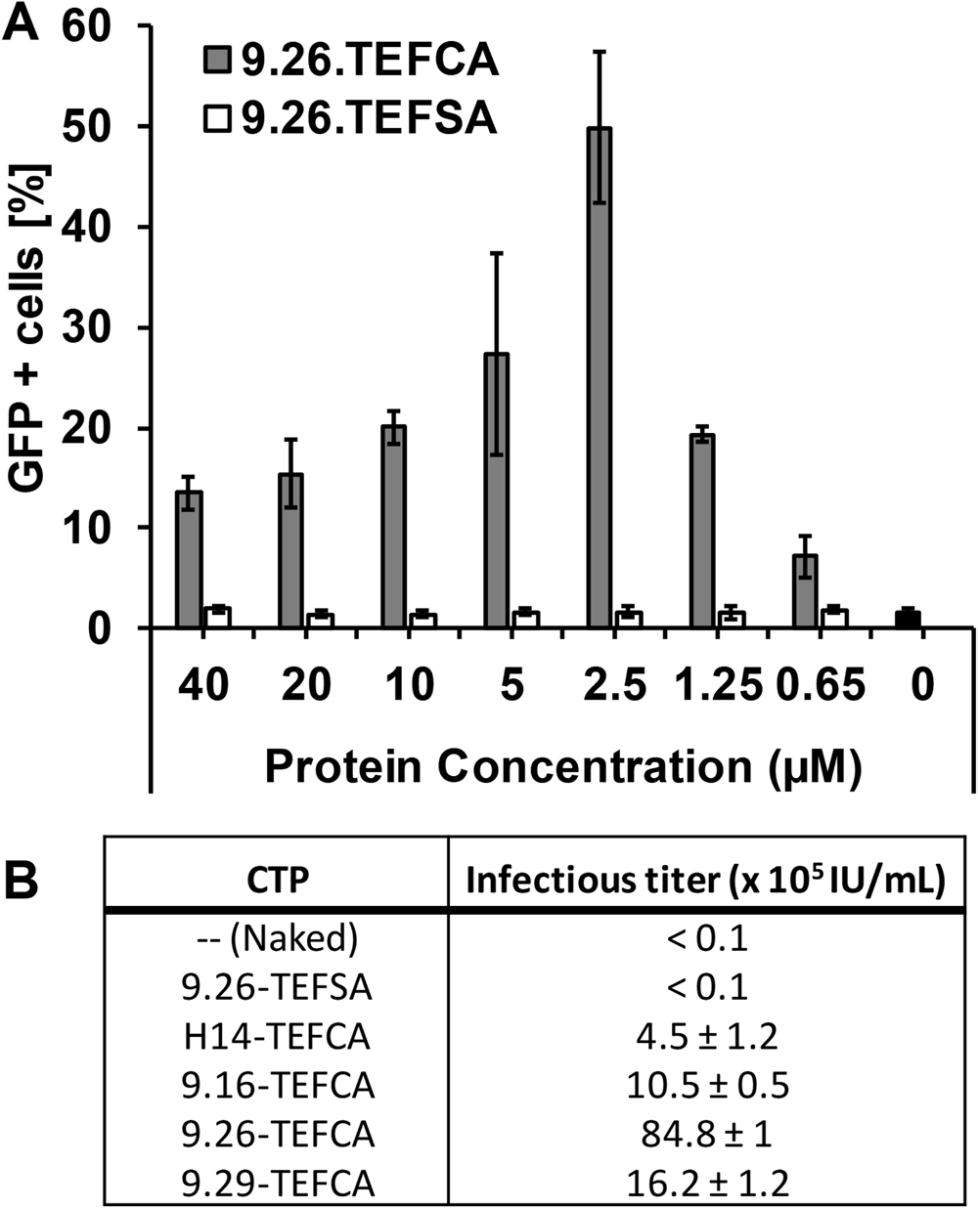
Transduction of HER2^+^ SKOV3 cells by DARPin-functionalized Sind-PDZ1-pp. (**A**) Bar diagram of the percentage of cells transduced with Sind-PDZ1-pp functionalized with different concentrations of DARPin.9.26-TEFCA or -TEFSA. The presence of intracellular GFP indicates successful transduction. (**B**) Infectious titer of Sind-PDZ1-pp functionalized with different DARPins (2.5 µM) in SKOV3 cells. Values and error bars represent the average and standard deviation, respectively, of two independent experiments.

We next carried out a dose response experiment to determine the ability of DARPin-displaying virions to transduce cells that express the target receptor. Given that DARPin.9.26 exhibited the highest disulfide-bond forming efficiency with PDZ1 (Fig. 2B) and was able to most efficiently retarget lentivirus pseudotyped with Sind-C* in our previous work^16^, we began our studies by determining the ability of DARPin.9.26-TEFCA to deliver Sind-PDZ1-pseudotyped lentivirus (Sind-PDZ1-pp, harboring a GFP reporter gene) to a specific target cell type. Sind-PDZ1-pp was incubated with increasing concentrations of DARPin.9.26-TEFCA or -TEFSA (negative control) at room temperature for 4 h, diluted 50-fold in OptiMEM medium and used to transduce HER2/neu^+^ ovarian cancer cell line SKOV3. As shown in Fig. 3A, DARPin.9.26-TEFCA was able to efficiently retarget Sind-PDZ1-pp in a dose dependent manner, with an optimal concentration of 2.5 µM (achieved >50% GFP^+^ cells). Reduced infectivity was observed when Sind-PDZ1-pp was incubated with higher concentrations of DARPin.9.26-TEFCA (e.g. >2.5 µM), likely resulting from the competition between the excess unbound DARPins and virion-associated DARPins for cell surface HER2-receptors. Only background transduction (~2% GFP^+^ cells) was observed for Sind-PDZ1-pp incubated with DARPin.9.26-TEFSA or the naked Sind-PDZ1-pp (0 µM), confirming that the specific interaction between the TEFCA pentapeptide and PDZ1 is needed for the targeted transduction.

To determine the infectious titer of the *in vitro* retargeted lentivirus, Sind-PDZ1-pp was incubated with 2.5 µM of different DARPin.X-TEFCA proteins at room temperature for 4 h, the mixtures were serially diluted and used to transduce SKOV3 cells. Unfunctionalized (naked) and DARPin.9.26-TEFSA (TEFSA) treated Sind-PDZ1-pp were included as controls. As shown in Fig. 3B, Sind-PDZ1-pp displaying DARPin.9.26-TEFCA exhibited the highest infectious titer (8.5 × 10^6^ IU/mL), >800-fold higher than that of the control naked Sind-PDZ1-pp (<10^4^ IU/mL) or Sind-PDZ1-pp incubated with DARPin.9.26-TEFSA (<10^4^ IU/mL). The infectious titer of our DARPin.9.26-TEFCA functionalized lentiviral vectors is >100-fold and >10-fold higher than that of lentiviruses in which the DARPins were displayed via fusion to an engineered measles virus envelope protein^24^ and Nipah virus envelope protein^13^, respectively. Lentiviruses functionalized with DARPin.9.16 and DARPin.9.29 achieved an intermediate infectious titer of 10^6^ and 1.6 × 10^6^ IU/mL, respectively, possibly in part due to the less efficient disulfide bond formation between these DARPins and PDZ1 (Fig. 2B). Interestingly, despite high disulfide bond forming efficiency, Sind-PDZ1-pp loaded with DARPin.H14R was the least infectious in SKOV3 cells with a titer of 4.5 × 10^5^ IU/mL (See discussion).

**Figure 4:**
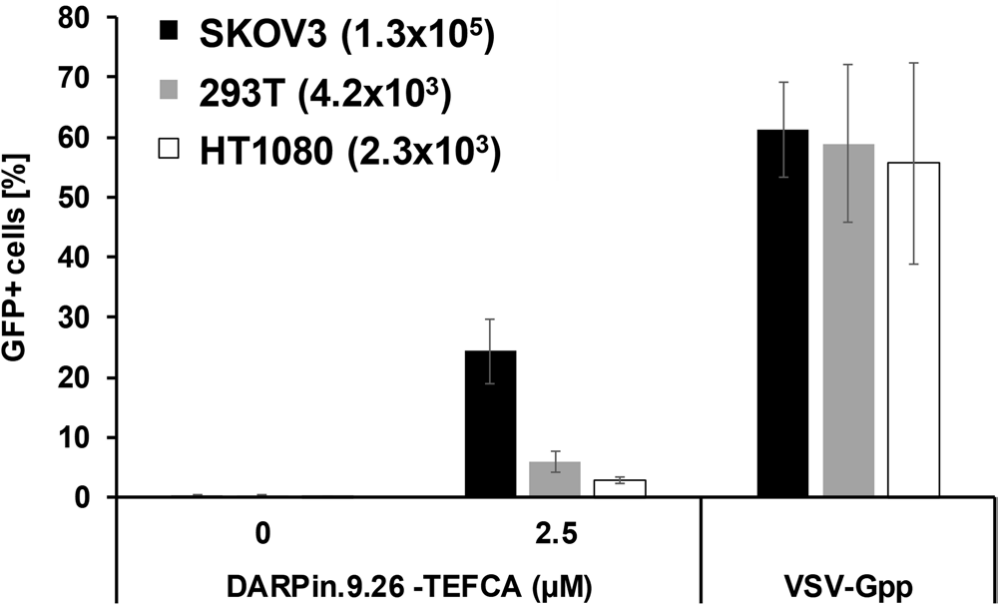
Transduction of DARPin.9.26-functionalized lentiviral vector is HER2-expression-level-dependent. Naked or DARPin-functionalized Sind-PDZ1-pp was used to transduce target cells with different levels of surface HER2 receptor (the estimated number of HER2 receptor per cell is shown in parenthesis^24^). Similar transduction efficiency was observed for VSV-Gpp in all cell types regardless of their cell surface HER2 levels.

As a further confirmation that the transduction activity of DARPin-loaded Sind-PDZ1-pp is mediated by the HER2 receptor, we compared the transduction efficiency of Sind-PDZ1-pp displaying DARPin.9.26-TEFCA across three cell lines displaying different surface levels of HER2 receptor (Fig. 4). As anticipated, the transduction efficiency generally correlates with the cell surface HER2 levels with higher efficiency transduction observed in cells displaying higher cell surface levels of HER2. Very low background transduction was observed in all cells transduced with the naked virus without DARPin functionalization. The positive control VSV-Gpp transduced all cell types with similar efficiency regardless of their cell surface HER2 levels.

### Lentiviral vectors retargeted by PDZ1 are stable under non-reducing conditions

**Figure 5:**
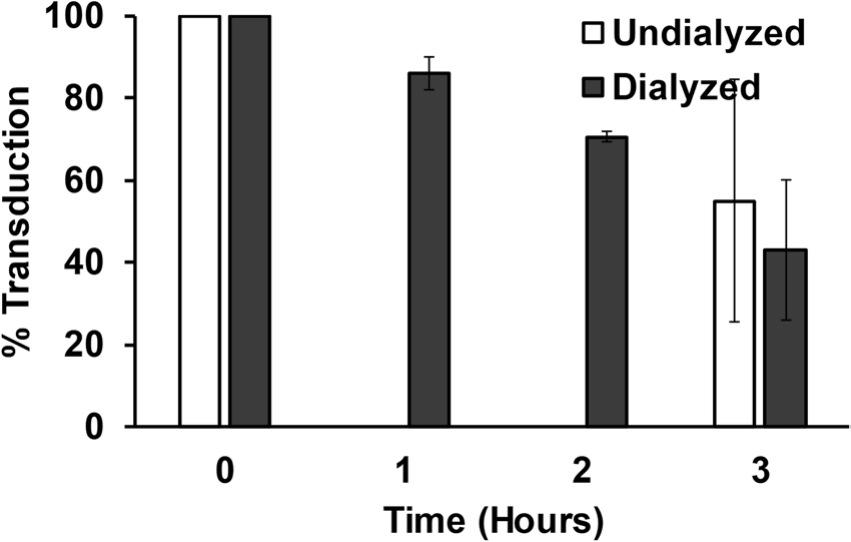
The PDZ1-TEFCA association is stable. Sind-PDZ1-pp functionalized with DARPin.9.26-TEFCA was dialyzed for 12 h at 4 °C to remove unbound DARPin.9.26-TEFCA prior to being divided into two aliquots. Aliquot 1 was continuously dialyzed at 4 °C for 3 days while aliquot 2 was stored at 4 °C without dialysis. The infectivity of these samples at the indicated times was measured in SKOV3 cells, and normalized to their corresponding values at Day 0. Values and error bars represent the average and standard deviation, respectively, of three independent experiments. The infectivity of undialyzed virions was quantified only on day 0 and day 3.

Since PDZ1 forms a covalent intermolecular disulfide bond with the TEFCA peptide^22^, the complex of PDZ1-TEFCA should be stable under non-reducing conditions, such as those observed in human serum, as redox environments of this type preserve the disulfide bond. To assess the stability of the retargeted lentivirus, Sind-PDZ1-pp was loaded with DARPin.9.26-TEFCA and then extensively dialyzed (MWCO 300 kDa) at 4 °C for >3 days. We reasoned that, if the functionalization is stable, we should observe minimum infectivity change between the dialyzed and undialyzed viruses. On the other hand, if the conjugation is not stable (reversible), any DARPin.9.26-TEFCA molecules that dissociate from the Sind-PDZ1-pp should be removed during the dialysis process, leading to reduced infectivity of the dialyzed virus compared to the undialyzed virus over time. In our previous study, the reversible interaction between the two halves of the split intein, despite of a low nanomolar K_d_, led to significantly reduced infectivity of the functionalized virions after 24 h of dialysis^16^. Sind-PDZ1-pp was incubated with DARPin.9.26-TEFCA (2.5 µM) at RT for 4 h, dialyzed at 4 °C for 12 h to remove unbound DARPin.9.26-TEFCA, and then divided into two samples; sample A was kept intact at 4 °C while sample B was further dialyzed at 4 °C. Aliquots from both samples were collected at different times and used to infect SKOV3 cells. As shown in Fig. 5, similar infectivity was observed between the dialyzed and undialyzed virus samples even after 3 days of continuous dialysis, confirming that the PDZ1-TEFCA complex can be used to stably functionalize pseudotyped lentivirus. The reduced infectivity seen in both virus samples is likely due to virion inactivation at 4 °C.

### Lentiviral vectors retargeted by PDZ1 are not inactivated by human serum complement

**Figure 6:**
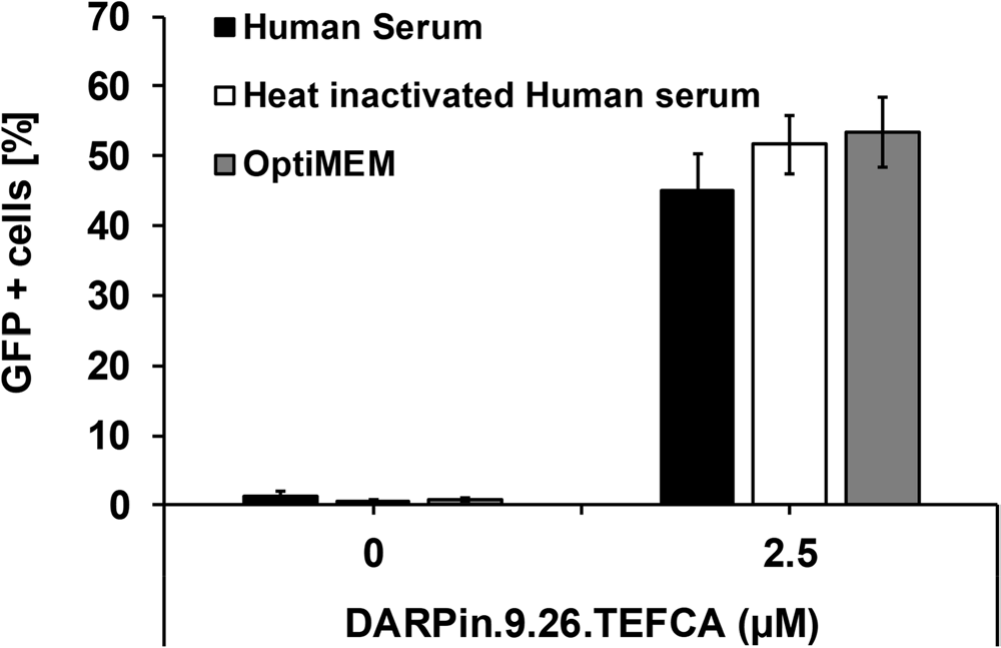
DARPin-functionalized Sind-PDZ1-pp is not inactivated by human complement. Sind-PDZ1-pp functionalized with DARPin.9.26-TEFCA were incubated with an equal volume of untreated human serum, heat-inactivated human serum or OptiMEM medium at 37 °C for 1 h. Each sample was diluted 50-fold with OptiMEM medium and used to infect SKOV3 cells. Values and error bars represent the average and standard deviation, respectively, of at least three independent experiments.

For *in vivo* application in humans, a gene therapy vector should not be inactivated by serum complement as these proteins attack pathogens by several mechanisms such as the lectin, classical and alternative pathways^26^. To investigate this, DARPin.9.26-TEFCA-displaying Sind-PDZ1-pp was mixed with an equal volume of either unmodified or heat-inactivated human serum and the mixtures were incubated at 37 °C for 1 h. The serum-exposed virus samples were then diluted 50-fold in OptiMEM medium and used to infect SKOV3 cells. As shown in Fig. 6, similar infectivity was observed for viruses incubated with untreated and complement-inactivated human serum, indicating that our retargeted lentiviral particles are not inactivated by human serum complement.

This result is consistent with (i) the finding that the Sindbis virus envelope proteins exhibit reduced sensitivity toward human serum^27^, and (ii) the functionalization moiety – PDZ1 – derives from Drosophila which is not known to be a human pathogen. The low serum sensitivity of lentiviruses retargeted using the PDZ1/TEFCA-mediated strategy is in contrast to (1) lentiviruses retargeted via pseudotyping with the engineered measles virus envelope protein^14,24,28^, for which the application is largely limited to measles virus vaccine-naïve patients, and (2) those retargeted via recruitment of cell-targeting antibodies through non-covalent interaction between Fc and the Fc-domain-specific ZZ domain from Protein A^18,20,27,29^, where serum immunoglobulins in immunocompetent individuals can potentially displace the conjugated antibodies^30^.

## Discussion

In this study, we developed a new modular platform for retargeting lentivirus through functionalization with a cell-targeting protein (CTP). A disulfide-bond forming protein-peptide pair, the N-terminal PDZ domain of InaD protein (PDZ1) and its pentapeptide ligand (TEFCA) from NorpA, was exploited as a molecular Velcro to retarget pseudotyped lentivirus to a desired cell type. The model CTPs in this study, HER2/neu-specific DARPins were fused to the N-terminus of TEFCA to be incorporated into lentiviruses pseudotyped with a receptor-blinded Sindbis virus envelope protein containing PDZ1 in an exposed extracellular loop. Unconcentrated Sind-PDZ1-pp functionalized with HER2/neu-specific DARPin.9.26-TEFCA efficiently transduced HER2^+^ SKOV3 cells with an infectious titer of 8.5 × 10^6^ IU/mL in SKOV3 cells, >800-fold higher than that of the unfunctionalized (naked) lentivirus (<10^4^ IU/mL). The infectious titer of these *in vitro* retarget lentiviral vectors in HER2^+^ SKOV3 cells is >100- and >10-fold higher than the reported titer in the same cells of lentiviruses on which the same DARPins were anchored via fusion to an engineered measles virus (MV) envelope protein^24^ and an engineered Nipah virus (NiV) envelope protein^13^, respectively. We believe that the high infectivity of our retargeted lentiviral vector is atleast in part due to the ability of the native Sindbis virus envelope protein to be efficiently incorporated into lentiviral virions. This observation is in contrast to that of both MV and NiV envelope proteins which require extensive engineering of the cytoplasmic region for incorporation into lentiviral particles^10,13,31^.

**Figure 7:**
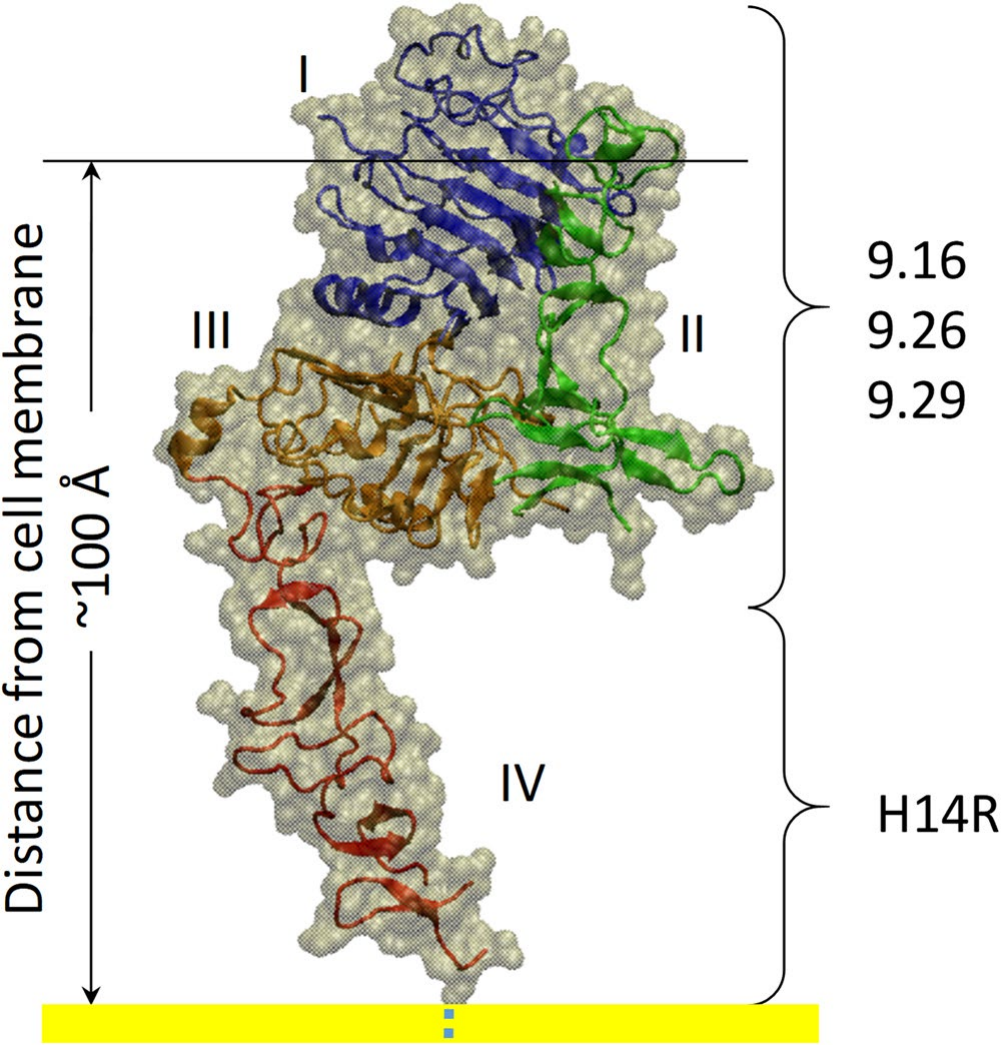
Three-dimensional structure of HER2/*neu* receptor (PDB ID: 1N8Z^36^) and the positions of the binding site of different DARPins relative to the cell membrane (yellow).

Functionalization of lentivirus with DARPins that bind different domains of HER2/neu receptor yielded very different infectious titers. Despite similar disulfide bond formation efficiency *in vitro* (Fig. 2), lentiviruses functionalized with DARPin.9.26-TEFCA (8.5 × 10^6^ IU/mL) exhibited >20-fold higher infectious titer in SKOV3 cells than those functionalized with DARPin.H14R (4.5 × 10^5^ IU/mL). This result is in contrast to a previous study in which DARPin.H14R-displaying Nipah virus (NiV) envelope protein pseudotyped lentivirus (NiV-pp) exhibited 100-fold higher infectivity than DARPin.9.26-displaying NiV-pp^13^. Virus entry requires the fusion between the viral and host membranes, necessitating that these membranes be brought sufficiently close to each other by the viral envelope protein and the cell surface receptor. The threshold distance required for viral fusion is expected to be viral envelope protein dependent and is predicted to be ~100 Å for NiV^13^. DARPin.H14R associates with domain IV of HER2, which is located closest to the cell membrane, while DARPin.9.16, 9.26 and 9.29 bind to domain I-III^23^ (Fig. 7). HER2/*neu* receptor Domain I and II are located at the tip of the receptor, farthest away from the cell membrane, while Domain III is in between domain IV and domain I/II.

In NiV-pp, the association of DARPin.H14R and domain IV likely reduced the distance between the viral and host membrane to below the threshold value (100 Å) and led to high transduction efficiency of DARPin.H14R-displaying NiV-pp. In contrast, the interaction between DARPin.9.26 and a more distal domain on HER2 was unable to do so, resulting in the lower transduction efficiency of DARPin.9.26-displaying NiV-pp relative to those displaying DARPin.H14R. The proximity of domain IV to the cell membrane also means that the viral protein needs to reach past domains I-III in order to interact with domain IV. In the NiV study, the different DARPins are tethered to the C-terminus of NiV-G protein via a flexible linker ((G_4_S)_3_), enabling these DARPins to freely access the cell receptor. In our study, the DARPins are fused to a TEFCA ligand via a short linker (G_4_) and are anchored onto the lentiviruses through interaction with PDZ1, which was inserted into a surface-exposed loop on the E2 glycoprotein of the Sindbis virus. Thus, the DARPins in our PDZ1/TEFCA tethering system may be unable to efficiently interact with Domain IV due to physical constraints, resulting in the reduced transduction efficiency of DARPin.H14R-functionalized Sind-PDZ1-pp. The high transduction efficiency achieved by DARPin.9.26-displaying virions in the PDZ1/TEFCA system also suggests that the minimum threshold distance required for Sindbis virus envelope protein-mediated viral fusion may be larger than that required for NiV envelope protein, enabling large receptor proteins to be used for targeting by lentiviral vectors.

The interaction between PDZ1 and the TEFCA penta-peptide is covalent and stable under non-reducing conditions (Fig. 2), a significant stability improvement upon our previous work which relied on high affinity, albeit non-covalent, protein-protein interaction between a pair of split intein for the conjugation of CTP to virions^16^. Previous efforts at tethering cell-specific monoclonal antibodies to the lentiviral surface via the association with antibody-Fc-domain-specific ZZ domain from Protein A were also vulnerable to the same stability limitations^18,20,27,29^. The reversible interaction between the ZZ domain and the antibody Fc domain makes this strategy particularly unsuitable for use in immuno-competent individuals in which serum immunoglobulins can compete with the conjugated antibody for binding to the ZZ domain. Finally, unlike lentivirus pseudotyped with VSV-G^5^, lentivirus displaying CTP tethered via the PDZ1/TEFCA interaction was insensitive to serum complement (Fig. 6), pointing to the potential of these viral vectors for *in vivo* applications.

In summary, we developed a simple lentivirus retargeting strategy that enables the creation of lentiviral vectors targeting essentially any cell types by covalent conjugation of specific CTPs *in vitro*. This strategy provides a path to the creation of a wide range of cell-specific lentiviral vectors for both research and clinical applications.

## Methods

### Cells and chemicals

HEK 293 T cells were purchased from Invitrogen (Carlsbad, CA). SKOV3 and HT1080 cells were kindly provided by Christian Buchholz (Paul-Ehrlich Institut; Langen, Germany)^24^. All cell lines were grown in Dulbecco’s Modified Eagle’s medium (DMEM) containing 4,500 mg/liter glucose, 4.0 mM glutamine, and 110 mg/liter sodium pyruvate (Thermo Scientific HyClone) supplemented with 10% fetal bovine serum (Atlanta Biologicals, Lawrenceville, GA) and 1× non-essential amino acids (Thermo Scientific HyClone). Dulbecco’s phosphate-buffered saline (DPBS) was purchased from Thermo Scientific HyClone.

### Lentivirus production

Pseudotyped lentiviruses were generated by co-transfecting 293 T cells with plasmids encoding (1) HIV gag-pol^32^, (2) pTRIP-eGFP^16^ and (3) the appropriate envelop protein at a 1:1:4 weight ratio using the TransIT reagent (Mirus Bio LLC, Madison, WI). The supernatants containing the pseudotyped lentiviruses were collected 48 h later, filtered (0.22 μm pore size) and stored at −80 °C in aliquots.

### Cell surface expression

To confirm cell surface expression of chimeric envelope proteins, 1.6 × 10^6^ HEK 293 T cells were transfected with 960 ng of the appropriate plasmid using Trans IT (Mirus Bio LLC; Madison, WI) as per the manufacturer’s protocol. Forty-eight hours post transfection; cells were harvested, washed and stained with a 1:1000 dilution of mouse anti-Flag (Genscript; Piscataway, NJ) in DPBS supplemented with 1% bovine serum albumin (BSA) for 1 h. These cells were then washed and stained with a 1:500 dilution of Dylight 488 goat anti-mouse (Jackson ImmunoResearch Laboratories, Inc; West Grove, PA) for 30 min. After removal of excess antibody, cells were resuspended in DPBS containing 1% paraformaldehyde (PFA) and analyzed using a BD FACSCalibur flow cytometer (BD Biosciences; San Jose, CA).

### Incorporation of chimeric envelope proteins

Lentiviruses pseudotyped with Sind-PDZ1 or Sind-C* were harvested and concentrated by ultracentrifugation (90 min; 40000 × g; 4 °C). The pellets were resuspended in 1/100^th^ original volume of DPBS and then mixed with 2X SDS loading buffer (0.5 M Tris-HCl, pH 6.8, 20% glycerol, 10% w/v SDS, 0.1% w/v bromophenol blue, 2% β-mercaptoethanol). Samples were boiled for 5 min at 95 °C, resolved on a 12% SDS-PAGE gel and electrotransferred onto a polyvinylidene difluoride (PVDF) transfer membrane (Pall Corporation; Pensacola, FL). Immunoblot analysis was performed with mouse anti HIV-1 p24 (1:250, NIH AIDS Reagent Program, Division of AIDS, NIAID, NIH: Monoclonal Antibody to HIV-1 p24 (No. 71-31) from Dr. Susan Zolla-Pazner)^33^ or mouse anti-Flag (1:1000, Genscript; Piscataway, NJ) and horseradish peroxidase-conjugated goat anti-mouse (Jackson ImmunoResearch; West Grove, PA) antibody. Protein bands were visualized by chemiluminescence using a ChemiDoc-It imager (UVP, LLC; Upland, CA).

### Protein expression and purification

All DARPin constructs were expressed in *Escherichia coli* BL21 (DE3) cells and purified by gravity Ni-NTA column as described previously^16^. Protein sequences for all the constructs are provided in the Supplementary Materials. Purified protein was concentrated to ~20–30 mg/ml using ultra-filtration spin columns (MWCO 10 kDa, Amicon Ultra, Millipore; Billerica, MA), dialyzed overnight against lysis buffer (10 mM Tris, 500 mM NaCl, pH 8.0) and stored at −80 °C until use.

### *In vitro* conjugation assay

Purified DARPin.X-TEFCA/TEFSA and PDZ1 proteins were mixed at a 1:1 molar ratio and incubated for 4 h at 25 °C. The protein samples were then mixed with an equal volume of 2X SDS sample buffer with or without 2% 2-mercaptoethanol (β-ME) and boiled for 5 min prior to resolution on a 12% SDS-PAGE gel. The amount of unreacted PDZ1 in each lane was quantified using the Trace Quantity module in Quantity One Software (BioRad, Hercules, CA, USA) and used to calculate the conjugation efficiency between different DARPin.X-TEFCA and PDZ1.

### Infection assays

Lentiviruses pseudotyped with Sind-PDZ1 (Sind-PDZ1-pp) were incubated with DARPin.X-TEFCA or DARPin.9.26-TEFSA at room temperature (RT) for 4 h. The mixture was then diluted 50-fold in OptiMEM medium and used to transduce SKOV3, HT1080 or 293 T cells (seeded the previous day in 48 well plates at 3.3 × 10^4^ cells/well) via spinoculation (300 × g for 1 h at RT followed by incubation for 2 h at 37 °C). The cells were then washed to remove unassociated viruses and incubated at 37 °C/5% CO_2_. If necessary, control lentiviruses pseudotyped with VSV-G harboring the same GFP reporter gene was included. These cells were harvested 48 hours later and the percentages of transduced cells (GFP^+^) were analyzed via flow cytometry. Virus titers were determined as described previously^34^.

### Stability assay

Unconcentrated supernatants containing Sind-PDZ1-pp were incubated with 2.5 µM DARPin 9.26-TEFCA for 4 h at RT, diluted 10-fold in dialysis buffer (1X PBS supplemented with 10mM L-glutathione and 0.05% sodium azide), transferred into a Float-A-Lyzer G2 Dialysis Device (MWCO 300 kDa) and dialyzed against dialysis buffer at 4 °C for 12 h to remove excess unreacted DARPin 9.26-TEFCA. The dialyzed virions were then divided into two samples. One sample was kept at 4 °C and the other was subjected to continued dialysis at 4 °C. At each time point, the appropriate volume of virus from each sample was harvested, supplemented with BSA (2.7 mg/mL), and stored at −80 °C. After the last time point, the equivalent volume from each aliquot was used to transduce SKOV3 cells (3.3 × 10^4^ cells/well in 48-well plates) seeded the previous day.

### Serum complement assay

Undiluted Sind-PDZ1-pp was incubated with DARPin.9.26-TEFCA (2.5 µM) for 4 h at 25 °C to allow association, and then incubated with an equal volume of untreated or heat-inactivated human AB serum (Corning; Corning, NY) and incubated for 1 h at 37 °C. The serum-exposed pseudoparticles were then used to transduce SKOV3 cells after 50-fold dilution in OptiMEM medium.

## Electronic supplementary material

Supplementary Information
