# Supplementary material for: A simple strategy for retargeting lentiviral vectors to desired cell types via a disulfide-bond-forming protein-peptide pair
Source: Sci Rep. 2018 Jul 20;8:10990. doi: 10.1038/s41598-018-29253-5 (PMC6054614; doi:10.1038/s41598-018-29253-5)
Supplement: Supplementary file 1 — Supplementary Information [file 41598_2018_29253_MOESM1_ESM.pdf]

Supplementary Materials and Methods

A Simple Strategy for Retargeting Lentiviral Vectors to Desired Cell Types via a Disulfide-bond-forming Protein-peptide pair

Nagarjun Kasaraneni[a], Ana M. Chamoun-Emanuelli[a], Gus A. Wright[b], Zhilei Chen[a*]

[a] Department of Microbial Pathogenesis and Immunology, Texas A&M University Health Science Center, College Station, Texas 77843, USA;
[b] Department of Veterinary Pathobiology, Texas A&M University, College Station, TX 77843, USA

E-mail: zchen4@tamu.edu

Index

S1. Protein sequences

Plasmids.

Plasmids encoding HIV Gag-Pol and vesicular stomatitis virus (VSV) envelope protein were kindly provided by Charles Rice (Rockefeller University, NY)[1].

HER2-binding DARPins were provided by Andreas Plückthun (University of Zurich; Zurich, Switzerland)[2, 3]. DARPin sequences were PCR amplified, incorporating a C-terminal G4 linker and TEFCA or TEFSA coding DNA, and inserted into pET15b vector between NdeI and XhoI sites.

DARPin.9.16-TEFCA
6his-DARPin.9.16-*L*-**TEFCA**

MGSSHHHHHHSSGLVPRGSHMGSDLGKKLLEAARAGQDDEVRILMANGADVNAHDFHGLT
PLHLAAGMGHLEIVEVLLKNGADVNAVDTDGITLLHLAAYYGHLEIVEVLLKHGADVNAHDYAG
STPLHLAANTGHLEIVEVLLKNGADVNAQDKFGKTAFDISIDNGNEDLAEILQ*GGGG***TEFCA**

DARPin.9.26-TEFCA
6his -DARPin.9.26-*L*-**TEFCA**

MGSSHHHHHHSSGLVPRGSHMGSDLGKKLLEAARAGQDDEVRILMANGADVNAKDFYGITPL
HLAAAYGHLEIVEVLLKHGADVNAHDWNGWTPLHLAAKYGHLEIVEVLLKHGADVNAIDNAGKT
PLHLAAAHGHLEIVEVLLKYGADVNAQDKFGKTAFDISIDNGNEDLAEILQ*GGGG***TEFCA**

DARPin.9.29-TEFCA
6his -DARPin.9.29-*L*-**TEFCA**

MGSSHHHHHHSSGLVPRGSHMGSDLGKKLLEAARAGQDDEVRILMANGADVNAHDFYGITPL
HLAANFGHLEIVEVLLKHGADVNAFDYDNTPLHLAADAGHLEIVEVLLKYGADVNASDRDGHTP
LHLAAREGHLEIVEVLLKNGADVNAQDKFGKTAFDISIDNGNEDLAEILQ*GGGG***TEFCA**

DARPin.H14R-TEFCA
6his -DARPin.H14R-*L*-**TEFCA**

MGSSHHHHHHSSGLVPRGSHMGSDLGKKLLEAARAGQDDEVRILMANGADVNATDIHGHTPL
HLAAAMGHLEIVEVLLKNGADVNANDWRGFTPLHLAALNGHLEIVEVLLKNGADVNATDTAGNT
PLHLAAWFGHLEIVEVLLKNGADVNAQDKFGKTAFDISIDNGNEDLAEILQ*GGGG***TEFCA**

DARPin.9.26-TEFSA
6his -DARPin.9.26-*L*-**TEFSA**

MGSSHHHHHHSSGLVPRGSHMGSDLGKKLLEAARAGQDDEVRILMANGADVNAKDFYGITPL
HLAAAYGHLEIVEVLLKHGADVNAHDWNGWTPLHLAAKYGHLEIVEVLLKHGADVNAIDNAGKT
PLHLAAAHGHLEIVEVLLKYGADVNAQDKFGKTAFDISIDNGNEDLAEILQ*GGGG***TEFSA**

PDZ1
(11 to 107 Amino Acids of InaD)
Plasmid expressing PDZ1 was provided by Prof. John Sondek [4]

AGELIHMVTLDKTGKKSFGICIVRGEVKDSPNTKTTGIFIKGIVPDSPAHLCGRLKVGDR

ILSLNGKDVRNSTEQAVIDLIKEADFKIELEIQTFDK

Sind-PDZ1

Sind-PDZ1 was constructed by inserting gene encoding PDZ1 into the BstEII restriction sites (between residues 71 and 74 in E2 envelope protein) in 2.2-ZZ [5, 6]

BstEII-*Flag*-**PDZ1**-BstEII
VT*DYKDDDDK***AGELIHMVTLDKTGKKSFGICIVRGEVKDSPNTKTTGIFIKGIVPDSPAHLCGR LKVGDRILSLNGKDVRNSTEQAVIDLIKEADFKIELEIQTFD**KGVT

References

1. Evans MJ, von Hahn T, Tscherne DM, Syder AJ, Panis M, Wolk B, et al. Claudin-1 is a hepatitis C virus co-receptor required for a late step in entry. Nature. 2007;446(7137):801-5. PubMed PMID: 17325668.
2. Munch RC, Muhlebach MD, Schaser T, Kneissl S, Jost C, Pluckthun A, et al. DARPins: an efficient targeting domain for lentiviral vectors. Mol Ther. 2011;19(4):686-93. Epub 2011/01/13. doi: 10.1038/mt.2010.298. PubMed PMID: 21224833; PubMed Central PMCID: PMC3070099.
3. Steiner D, Forrer P, Pluckthun A. Efficient selection of DARPins with sub-nanomolar affinities using SRP phage display. J Mol Biol. 2008;382(5):1211-27. doi: 10.1016/j.jmb.2008.07.085. PubMed PMID: 18706916.
4. Kimple ME, Sondek J. Affinity tag for protein purification and detection based on the disulfide-linked complex of InaD and NorpA. BioTechniques. 2002;33(3):578, 80, 84-8 passim. PubMed PMID: 12238768.
5. Morizono K, Bristol G, Xie YM, Kung SK, Chen IS. Antibody-directed targeting of retroviral vectors via cell surface antigens. J Virol. 2001;75(17):8016-20. Epub 2001/08/03. PubMed PMID: 11483746; PubMed Central PMCID: PMC115045.
6. Pariente N, Morizono K, Virk MS, Petrigliano FA, Reiter RE, Lieberman JR, et al. A novel dual-targeted lentiviral vector leads to specific transduction of prostate cancer bone Metastases In vivo after systemic administration. Molecular therapy : the journal of the American Society of Gene Therapy. 2007;15(11):1973-81. doi: Doi 10.1038/Sj.Mt.6300271. PubMed PMID: ISI:000250382400017.
